# Integrated Polarization-Splitting Grating Coupler for Chip-Scale Atomic Magnetometer

**DOI:** 10.3390/bios12070529

**Published:** 2022-07-15

**Authors:** Jinsheng Hu, Jixi Lu, Zihua Liang, Lu Liu, Weiyi Wang, Peng Zhou, Mao Ye

**Affiliations:** 1 School of Instrumentation and Optoelectronic Engineering, Beihang University, Beijing 100191, China; hujins10@buaa.edu.cn (J.H.); liangzh@buaa.edu.cn (Z.L.); zb2117117@buaa.edu.cn (P.Z.); 2 Beihang Hangzhou Innovation Institute Yuhang, Xixi Octagon City, Yuhang District, Hangzhou 310023, China; by2143137@buaa.edu.cn (L.L.); weiyiwang@buaa.edu.cn (W.W.); maoye@buaa.edu.cn (M.Y.); 3 Research Institute for Frontier Science, Beihang University, Beijing 100191, China

**Keywords:** integrated photonics, grating coupler, FDTD, chip-scale atomic magnetometer, bio-magnetic imaging

## Abstract

Atomic magnetometers (AMs) are widely acknowledged as one of the most sensitive kind of instruments for bio-magnetic field measurement. Recently, there has been growing interest in developing chip-scale AMs through nanophotonics and current CMOS-compatible nanofabrication technology, in pursuit of substantial reduction in volume and cost. In this study, an integrated polarization-splitting grating coupler is demonstrated to achieve both efficient coupling and polarization splitting at the D1 transition wavelength of rubidium (795 nm). With this device, linearly polarized probe light that experienced optical rotation due to magnetically induced circular birefringence (of alkali medium) can be coupled and split into individual output ports. This is especially advantageous for emerging chip-scale AMs in that differential detection of ultra-weak magnetic field can be achieved through compact planar optical components. In addition, the device is designed with silicon nitride material on silicon dioxide that is deposited on a silicon substrate, being compatible with the current CMOS nanofabrication industry. Our study paves the way for the development of on-chip AMs that are the foundation for future multi-channel high-spatial resolution bio-magnetic imaging instruments.

## 1. Introduction

Atomic devices based on the light–atom interaction mechanism such as atomic clocks [1,2], atomic magnetometers [3,4], nuclear magnetic resonance gyroscopes [5,6], and laser frequency stabilizers [7,8] have experienced rapid development in recent decades. Motivated by urgent demand of multi-channel high-spatial resolution bio-magnetic imaging [9,10,11], micro positioning navigation-timing (MPNT) [12], ultra-low-field and zero-to-ultra low field nuclear magnetic resonance (NMR) [13,14,15,16], and chip integration of atomic devices has become an hot topic for researchers worldwide. While microfabricated vapor cells have attracted much attention [17,18,19,20], optical components manipulating the atomic state in vapor cell (optical pumping and detection) are still bulky and cumbersome (e.g., prism, polarizer, half/quarter wave plate, and polarization beam splitter). In addition, these traditional components are not compatible with nanofabrication technology, which has become major obstacle of chip integration and further miniaturization. On the other hand, the field of integrated photonics, especially silicon photonics, have evolved rapidly, enabling the integration of multi-functionally traditional optics on chip [21,22]. Numerous industrial platforms have been developed in the past few years to facilitate the fabrication of integrated photonics devices. Therefore, it is promising to achieve complete chip integration of atomic devices through emerging integrated photonics and CMOS-compatible nanofabrication technology [23,24,25], which promotes both drastic miniaturization and mass production.

Currently, there are two major schemes for the application of integrated photonics in realizing light–atom interaction. For the first scheme, atoms interact directly with the evanescent field in the vicinity of the guided optical modes [26,27,28]. In this scheme, a fraction of light that is determined by the guided mode (confined mode) interacts with atoms around the waveguide, which is embedded on the surface of vapor cell. Although the confined mode has the effect of enhancing light–atom interaction, its application is limited due to a small interaction region. For the second scheme, light is coupled in or out by couplers from a planar integrated photonic device to tridimensional space (vapor cell), aiming to achieve large-scale free-space light–atom interaction [29,30,31]. This scheme is straightforward and convenient to implement for conventional vapor cells. Thus, it is compatible with current atomic devices.

Among all atomic devices based on the light–atom interaction mechanism, atomic magnetometers (AMs) stand out for their ultra-high sensitivity (reaching sub-fT/Hz^1/2^ scale). AMs nowadays have wide applications including bio-magnetic imaging such as magnetoencephalography (MEG) [32,33,34] and magnetocardiography (MCG) [35,36]. AMs are classified into single-beam and double-beam configurations. Single-beam AMs typically apply a circularly polarized beam to achieve optical pumping and magnetic field detection simultaneously [37,38]. Among double-beam AMs, there exists cases where the pump and probe beam cross perpendicularly, co-propagating or nearly co-propagating. For the case where pump and probe beam cross perpendicularly, it is the most optimal in terms of sensitivity [39,40,41,42]. For the case where pump and probe beams co-propagate, compared with the first case, this configuration is particularly for miniaturization, but the sensitivity is sacrificial [43,44]. As for near co-propagation of pump and probe beams, this is a compromise between the perpendicular and co-propagating cases. The pump and probe beams cross at a small angle at the vapor cell to allow for independent spatial detection of the probe beam only [45,46]. Moreover, it is particularly fascinating for miniaturization of AMs by means of single elliptically polarized beam; the circularly polarized component is utilized to polarize the alkali-metal atoms, while the linearly polarized light is utilized to detect optical rotation angle due to magnetic field [47,48]. The atomic magnetometer (AM) operates with net spin polarization of alkali atoms through optical pumping. This process can be modified by external magnetic field, and the spin polarization undergoes Larmor precession. The new procession state, which is a function of the external magnetic field, changes the absorptive and dispersive properties of the atomic medium. Then, information of the external magnetic field can be detected by circularly polarized pump light (mostly in single-beam AM) or linearly polarized probe light (mostly in double-beam AM) transmitting through the atomic medium. Traditional and bulk PBS used for optical rotation angle detection are cumbersome, wherein its integration in miniaturized AMs becomes extremely difficult. Moreover, this is the main reason that conventional miniaturized AMs mostly utilize single-beam configuration by means of the optical absorption method. Compared with the optical rotation angle differential detection, the optical absorption method is affected by common mode factors such as light intensity fluctuations, which greatly limit the performance of miniaturized AMs. Therefore, it is a very challenging problem to implement differential detection in chip-scale AMs. In the past two years, there have been some demonstrations of chip-scale AM utilizing the second scheme to achieve differential detection: Sebbag et al. developed a nanoscale photon spin sorter (PSS) to measure the polarization of the linearly probe light [49]. However, there exists a spot size mismatch between the PSS structure and incident light, so the coupling efficiency is comparatively low; Yang et al. demonstrated a chip-scale AM using metasurface-based nanophotonic components including waveplate and polarizing beamsplitter [50], while silicon is not transparent in D1 transition wavelength of rubidium (795 nm) and its structure is difficult to fabricate due to a number of nanoscale elliptical posts, which became non-negligible drawbacks.

Generally, it is challenging to realize high-efficiency coupling and polarization splitting of linearly polarized probe light (e.g., differential detection of magnetic field) in chip-scale AM. To overcome this obstacle, in this study, we developed a polarization-splitting grating coupler that achieved efficient coupling and polarization-splitting at the D1 transition wavelength of Rb (795 nm). This device is designed with silicon nitride on silicon dioxide, which is deposited on silicon substrate. Compared with the traditional bulky polarimetry system, our device enables dramatic reduction of volume while facilitating mass production through the current CMOS-compatible nanofabrication platform. Moreover, this device is especially advantageous for future on-chip multi-channel AMs, which is in urgent demand for multi-channel high-spatial-resolution MEG and MCG.

## 2. Materials and Methods

### 2.1. Numerical Analysis of Magnetic Field Measurement

In order to illustrate application of designed polarization-splitting grating coupler, in this paper, SERF AM (^87^Rb, modulated field) with a single elliptically polarized beam nearly resonant with D1 transition of ^87^Rb is represented in an instance [47,48]; in practice, this device is capable of being utilized in a variety of AMs. The mechanism of magnetic field sensing is based on magnetically induced circular birefringence in atomic medium [51]. The linearly polarized probe light is nearly resonant with the D1 line of Rb and propagate parallel to the circularly polarized pump beam and its polarization rotates by an angle θ due to a difference in real part indices of refraction $n_{+}(\nu )$ and $n_{-}(\nu )$ experienced by $\sigma^{+}$ and $\sigma^{-}$ light, respectively. The θ is given by [47,52]

(1) $$\theta =\frac{\pi \nu l}{c}[n_{+}(\nu )-n_{-}(\nu )]$$

The real part indices of refraction are as follows:

(2) n+(ν)=1+(nrec2fD14ν)(1−Pz)Im[L(ν−νD1)]

(3) n−(ν)=1+(nrec2fD14ν)(1+Pz)Im[L(ν−νD1)]

where l is length of vapor cell, n is density of alkali vapor, $r_{e}$ is classical electron radius, c is speed of light, $f_{D1}$ is oscillator strength of the D1 transition of ^87^Rb, is frequency, and $\nu_{D1}$ is D1 resonance frequency. $P_{z}$ is projection of spin polarization along the *z*-direction. Im[L(ν−νD1)] is the imaginary part of the complex Lorentzian curve. Thus, the value of rotation angle θ is given by

(4) θ=π2nlrecPzfD1Im[L(ν−νD1)]

Under the condition of high alkali vapor density and near-zero magnetic field (SERF regime), the spin-exchange rate is sufficiently larger compared to the atomic Larmor precession frequency. The equilibrium state of atomic spin can be well characterized by a spin-temperature distribution; in this condition, the complicated density matrix equation can be simplified to the phenomenological Bloch equation [28]:

(5) $$\frac{d\vec{P}}{dt}=\frac{1}{Q(P)}[\gamma \vec{P}\times B+R_{op}(s-\vec{P})-R_{rel}^{tot}\vec{P}]$$

where $\vec{P}$ is electron spin polarization of alkali atoms, Q(P) is nuclear slow-down factor that depends on the spin polarization, γ is gyromagnetic ratio of bare electron, $\vec{B}$ is external magnetic field including static and oscillating components, $R_{op}$ is optical pumping rate, s is photon spin of incident light, and $R_{rel}^{tot}$ is total intrinsic transverse relaxation rate of electron spin polarization. In this study, we considered the unknown static magnetic field along the *x*-direction, and a modulated magnetic field ($B_{x}^{tot}=B_{x}+B_{mod}\cos(\omega t)$) was applied to facilitate precise measurement of $B_{x}$ by shifting the detected signal to a higher modulation frequency ω, which avoids low-frequency noise such as the vibration of a laser beam. In this condition, the analytic solution of the Bloch equation can be acquired, and a component of $P_{z}$ is shown as follows:

(6) $$P_{z}(\omega )=2sR_{op}J_{0}(\alpha )J_{1}(\alpha )\frac{\gamma B_{x}}{{\left(\gamma B_{x}\right)}^{2}+R^{2}}\sin(\omega t)$$

where $\alpha =\gamma B_{mod}/Q(P)\omega$ is the modulation index, $R_{op}+R_{rel}^{tot}=R$, $J_{0}$ and $J_{1}$ are Bessel functions of the first kind; then, the signal is demodulated by a lock-in-amplifier at first harmonic:

(7) $$P_{z}(B_{x})=2sR_{op}J_{0}(\alpha )J_{1}(\alpha )\frac{\gamma B_{x}}{{\left(\gamma B_{x}\right)}^{2}+R^{2}}$$

Eventually, the function between the optical rotation angle θ and static unknown magnetic field $B_{x}$ is concluded as follows:

(8) κ(ν)=πnlrecfD1sRopJ0(α)J1(α)Im[L(ν−νD1)]

(9) $$\theta (B_{x},\nu )=\kappa (\nu )\frac{\gamma B_{x}}{{\left(\gamma B_{x}\right)}^{2}+R^{2}}$$

It can be seen from Equation (9) that when the detuning frequency ($\Delta =\nu -\nu_{D1}$) of the linearly polarized probe light is determined, the optical rotation angle θ is a single-valued function of external magnetic field $B_{x}$. Equation (9) describes a typical Lorentzian profile, and there is an approximate linear area near zero-field, which is typical measurement range of the AM. To make the photodetector difference signal monotonic as a function of the unknown static magnetic field $B_{x}$, operating parameters of AM such as modulation index α should be chosen judiciously to allow the optical rotation angle θ to vary within π/2 [47].

### 2.2. Materials and Structure of the Polarization-Splitting Grating Coupler with Horizontal Integration

**Figure 1:**
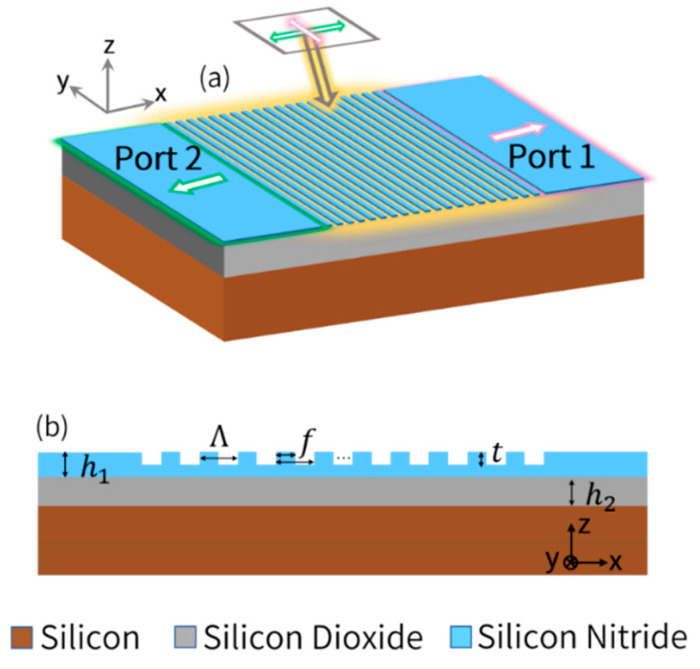
(**a**) Sketch of the proposed polarization-splitting grating coupler, which can spatially separate orthogonal linear-polarized lights to Port 1 and Port 2. (**b**) Detailed cross-section view of the grating coupler.

**Figure 2:**
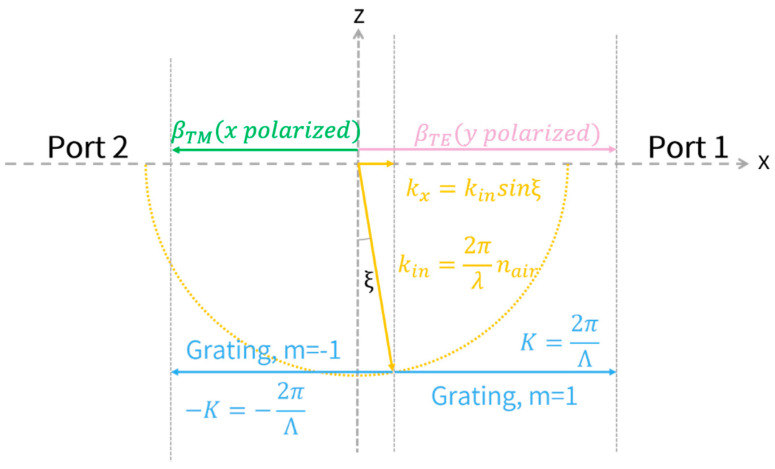
Graphical illustration of the Bragg condition satisfied by the polarization-splitting grating coupler.

The overall structure of polarization-splitting grating coupler is shown in Figure 1a. The refractive indexes of silicon (Si) and silicon dioxide (SiO_2_) in the material database are 3.711 and 1.453 at 795 nm, respectively. Silicon nitride (SiN) with a thickness of 260 nm is applied instead of common SOI (silicon-on insulator) wafer as guided layer due to low propagation loss and suitable refractive index at λ = 795 nm (*n_0_ =* 1.997), which is the D1 transition wavelength of Rb. In addition, the transparent window of SiN extends to the visible and the near infrared spectrum, which can be applied for interaction of light with alkali atoms (e.g., K, Rb, and Cs) [53]. The cross-section view of this structure is depicted in Figure 1b. The two dimensional finite-difference-time-domain (FDTD) method is utilized to simulate the effectiveness of our design due to its broad width (12 μm) [54]. Non-uniform mesh grids are utilized in order to obtain trade-off between sufficient simulation accuracy and simulation time. Perfectly matched layer (PML) is applied in all directions to avoid boundary reflection. The grating operation can be well comprehended in terms of the Huygens–Fresnel principle, namely, constructive and destructive interference arising from wavefronts, which are generated by the diffraction of light from the grating teeth and grooves. The polarization-splitting grating coupler proposed in this paper is well described by the Bragg Law [55], as shown in Figure 2. The wave incident on grating is a guided wave propagating in a slab waveguide, with a direction of propagation in the identical plane as the grating, and is normal to the grating teeth. Grating coupler is a polarization-sensitive device, which means that incident light with different polarization (x-polarized or y-polarized) will excite different guided modes in the slab waveguide [56]. An additional compensatory wave vector is introduced by grating structure (blue arrow shown in Figure 2), which enables matching of the incident light wave vector ($k_{in}$ shown in Figure 2) and fundamental guided mode wave vector in the waveguide ($\beta_{TE}$ and $\beta_{TM}$ shown in Figure 2), and thus the fundamental transverse electric (TE) and fundamental magnetic (TM) mode are excited in Port 1 and Port 2, respectively, as described in Equations (10) and (11):

(10) $$\mathrm{Port}\;1\;(y\text{-}\mathrm{polarized}):\;\beta_{TE}=k_{in}\sin\xi +2\pi /\Lambda$$

(11) $$\mathrm{Port}\;2\;(x\text{-}\mathrm{polarized}):\;\beta_{TM}=k_{in}\sin\xi -2\pi /\Lambda$$

where $\beta_{TE}$ and $\beta_{TM}$ are propagation wave vector of the excited mode (i.e., fundamental TE and TM mode) in slab waveguide. $k_{in}$ is the incident wave vector, K=2π/Λ is the reciprocal lattice vector of the grating (i.e., additional compensatory wave vector), Λ is the grating period, and ξ is the tilted angle of incident light. In Figure 2, *m* is the diffraction order, where m=1 for fundamental TE mode and m=−1 for fundamental TM mode are utilized in this paper. The calculated results of grating parameters are indicated in latter sections. When incident light with proper tilted angle propagates to the grating coupler, two orthogonal components (x- and y-polarized) are spatially divided and propagate to contrary directions, which are then coupled into two separate planar waveguides (Port 1 and Port 2 shown in Figure 1a). Specifically, the y-polarized component will be coupled to the fundamental TE mode of the high aspect ratio planar waveguide (12 μm × 260 nm, Port 1), while the x-polarized component will be coupled to the TM mode of the planar waveguide (Port 2) [57]. In order to suppress the effect of fluctuant intensity of incident light on measurement results and improve signal-to-noise ratio (SNR), differential detection of Port 1 and Port 2 is applied to measure the optical rotation angle of the linearly polarized probe light.

As described above, the polarization-splitting grating coupler is designed to couple and split linearly polarized probe light that experienced optical rotation due to magnetically induced circular birefringence. However, it can be noticed that the high aspect ratio cross-section of the planar waveguide is 12 μm × 260 nm, which has a large mode mismatch with lensed fiber and cannot be directly coupled [58,59]. In order to efficiently extract the differential signal that is used to measure the optical rotation angle, a high-efficiency fiber-chip spot size converter is needed to serve as a coupler to achieve mode matching between the lensed fiber with a mode field diameter (MFD) of about 4 μm and high aspect ratio mode profile of planar waveguide (12 μm × 260 nm). In this work, we demonstrated a horizontal integration of the polarization-splitting grating coupler that consists of a mode converter, a single-mode strip waveguide and an inverse taper, which is capable of coupling light from planar waveguide input to lensed fiber output efficiently at a D1 transition wavelength of Rb (795 nm).

**Figure 3:**
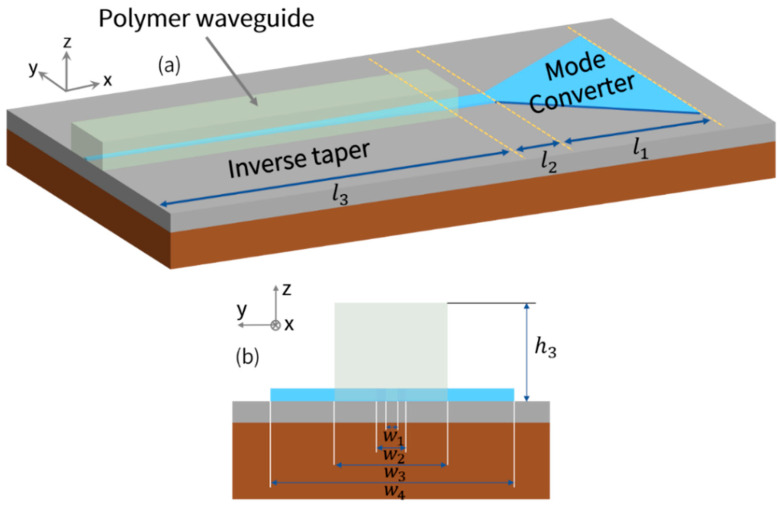
(**a**) Schematic illustration of our proposed fiber-chip spot size converter (horizontal integration), which consists of a mode converter, single-mode strip waveguide, and inverse taper. (**b**) Detailed cross-section of the considered structure near the chip facet.

A schematic illustration of our proposed fiber-chip spot size converter (i.e., horizontal integration) is shown in Figure 3. Mode transition between the high aspect ratio planar waveguide and highly confined single-mode stripe waveguide is performed by the mode converter, and then the inverse taper is utilized to couple light from strip waveguide to lensed fiber. The length of the mode converter, single-mode stripe waveguide, and inverse taper are marked by $l_{1}$, $l_{2}$ (=5 μm), and $l_{3}$, respectively, as displayed in Figure 3a. A cross-section of the horizontal integration where the width of inverse taper tip, single-mode stripe waveguide, and planar waveguide are denoted by $w_{1}$, $w_{2}$, and $w_{4}$, as shown in Figure 3b. A 5 μm × 5 μm ($w_{3}$ = $h_{3}$ = 5 μm) polymer waveguide with a refractive index of about 1.506 covers the inverse taper, as shown in Figure 3a. The polymer waveguide is capable of mode size and effective refractive index matching between lensed fiber and chip, which means it is able to efficiently guide light from the inverse taper to lensed fiber [60]. Three-dimensional eigenmode expansion (3D-EME) and finite difference eigenmode (FDE) methods are exploited to design the horizontal integration, aiming to achieve high-efficiency coupling between the high aspect ratio planar waveguide (Port 1 and Port 2) and lensed fiber for both fundamental TE and TM modes at 795 nm.

## 3. Simulation Results and Discussions

### 3.1. Characterization of Polarization-Splitting Grating Coupler

**Figure 4:**
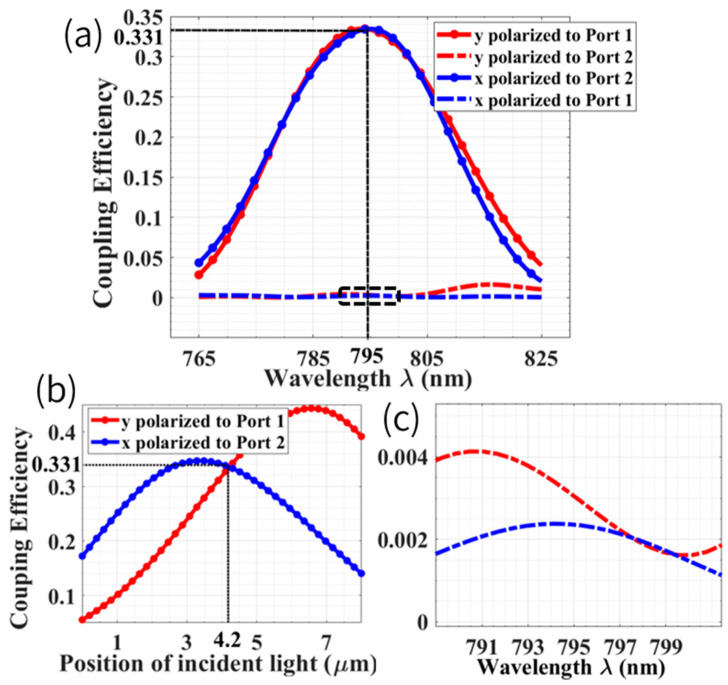
(**a**) Simulated coupling efficiency versus wavelength of incident light. (**b**) The dependence of coupling efficiency as a function of incident light position; zero at *x*-axis means left boundary of grating. (**c**) The enlarged view inside the dotted box in (**a**).

A Gaussian beam with a diameter of 8 μm was applied as the incident light of the grating coupler. Thickness of SiN ($h_{1}$) was 260 nm, grating fill factor (f) was 0.5, grating thickness (t) was 100 nm, and number of periods was 20. To satisfy the Bragg condition and maximize coupling efficiency [61], the grating period (Λ) and thickness of silicon dioxide ($h_{2}$) were 485 nm and 2025 nm, respectively. In addition, the tilted angle of incident light was 4° to minimize back reflection. Figure 4 shows the simulated coupling efficiency of orthogonal linear-polarized components (coupled to Port 1 and Port 2, respectively) under variation of incident wavelength and position. Figure 4a,b shows that when incident light deviates 4.2 μm from the left boundary of grating, the coupling efficiency was 33.1% at 795 nm for both x and y linearly polarized lights. Figure 4b reveals that maximal coupling efficiencies for y-polarized light at Port 1 and x-polarized light at Port 2 were 44.3% and 34.7%, respectively. Polarization-induced extinction ratio (PIER) was calculated to evaluate the crosstalk of different ports, which is defined as

(12) $$PIER_{x}=10\log_{10}\frac{I_{Port2}^{x}}{I_{Port1}^{x}};PIER_{y}=10\log_{10}\frac{I_{Port1}^{y}}{I_{Port2}^{y}}$$

where $PIER_{x}$ and $PIER_{y}$ are polarization-induced extinction ratio of x- and y-polarized light, respectively.$I_{Port1}^{x/y}$ and $I_{Port2}^{x/y}$ are intensity of x- and y-polarized components in Port 1 and Port 2, respectively. Both $PIER_{x}$ and $PIER_{y}$ exceeded 20 dB, as shown in Figure 4a,c. It is worth mentioning that compared to coupling efficiency, polarization selectivity (i.e., high PIER) was more preferable because differential detection was applied in our configuration.

**Figure 5:**
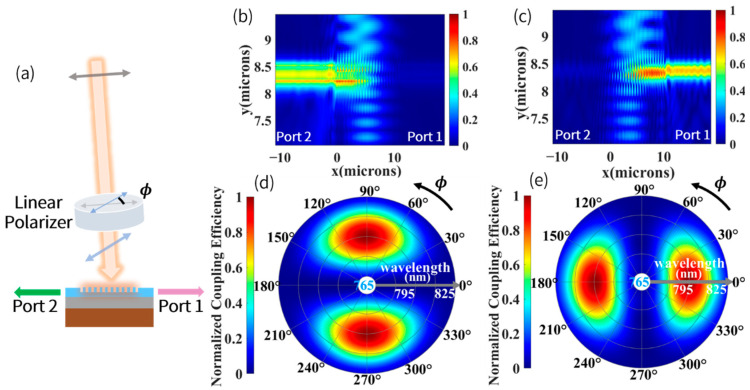
(**a**) Sketch of setup of the grating coupler for testing function of measuring linearly polarized angle ϕ. (**b**) Electric field distribution along the grating structure when incident light was x-polarized. (**c**) When incident light was y-polarized. (**d**) Simulated normalized coupling efficiency as a function of polarizer rotation angle ϕ and wavelength of the incident light in Port 1. (**e**) In Port 2.

In order to acquire the signal response of our device under different incident polarization angle ϕ, the grating coupler was simulated by focusing the incident light with variated linearly polarized angle ϕ, as shown in Figure 5a. When incident light was x-polarized, wherein the electric field distribution is shown in Figure 5b, it was evident that light was coupled to Port 2, while some of light leaked into the substrate. Figure 5c shows electric field distribution along the grating structure when incident light was y-polarized. The normalized coupling efficiency as a function of polarizer rotation angle ϕ is depicted in Figure 5d,e. It is shown that the linearly polarized angle ϕ could be obtained by a differential signal in Port 1 and Port 2, but it should be mentioned that only change of polarized angle within π/2 can be accurately calculated due to periodicity of the differential signal. It is necessary to note that variation of the optical rotation angle with external magnetic field can be precisely tuned by controlling parameters in AM, so with proper parameters selected, which is discussed in Section 2.1, the external magnetic field was measured accurately in the near zero-field range.

### 3.2. Design and Optimization of Fiber-Chip Spot Size Converter

**Figure 6:**
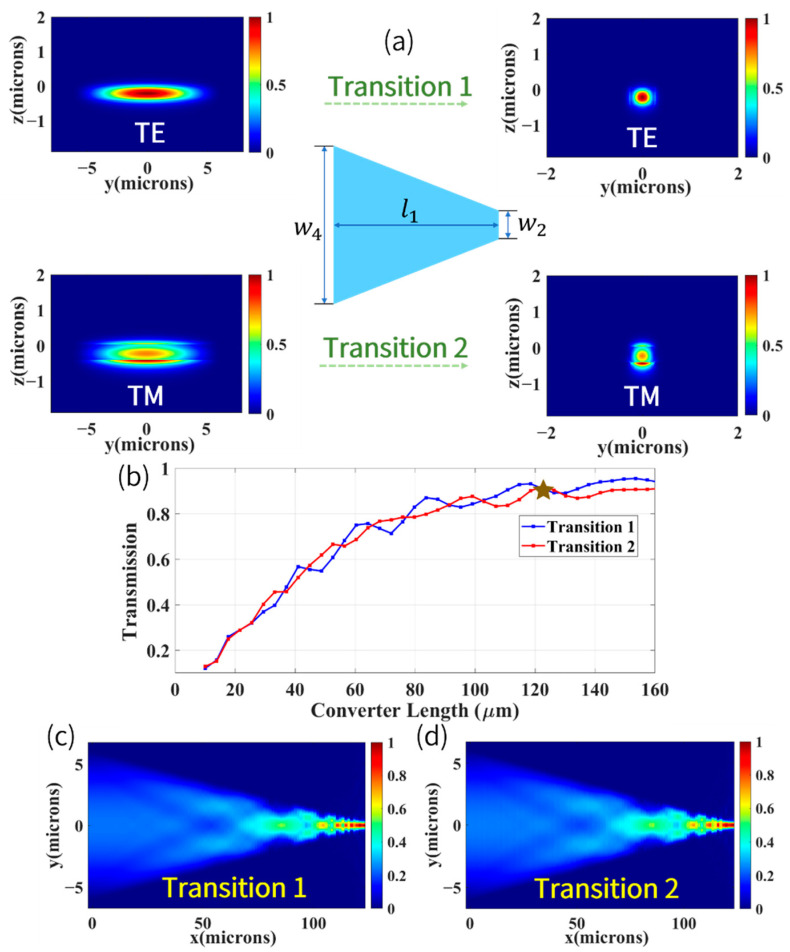
(**a**) Top view of the mode converter and its functionality. (**b**) Transmission of the Transition 1 and Transition 2 as a function of length of the mode converter. (**c**) Simulated electric field profile for Transition 1 at a wavelength of 795 nm. (**d**) For Transition 2.

In order to make the balanced-polarimetric readout system for chip-scale AM straightforward, compared with adiabatic taper, the common linear taper was applied to achieve polarization-independent efficient mode conversion. Figure 6a illustrates the functionality of the linear mode converter connecting a 12 μm wide input waveguide ($w_{4}$) and a 0.5 μm wide single-mode stripe waveguide ($w_{2}$). It enables conversion of fundamental TE mode (Transition 1) and TM mode (Transition 2) of two waveguides with different cross-sections while experiencing relatively little mode conversion to higher-order modes. From Figure 6b, we can see that with the increase in converter length ($l_{1}$), the transmission (i.e., coupling efficiency) ascended gradually, and the transmission of both TE and TM transition reached 91.5% when $l_{1}$ was equal to 121.5 μm, as marked by the brown star in Figure 6b. As the converter length increases to several hundred microns, the transmission of Transition 1 and Transition 2 will continue to improve. Because differential detection was harnessed in our work, it is more necessary to achieve relatively high-efficiency coupling with the same efficiency of TE and TM modes, rather than pursuing 100% conversion efficiency, and it is reasonable to determine the length as 121.5 μm from the perspective of compact structure. In Figure 6c,d, we show the numerical calculation of the electric field profile of Transition 1 and Transition 2 at a wavelength of 795 nm. It is noticeable that most of the fundamental modes (TE and TM) of the high aspect ratio planar waveguide were converted to the fundamental modes of the single-mode strip waveguide, and indications that a small amount of mode conversion from the fundamental modes to the higher-order modes can also be observed.

**Figure 7:**
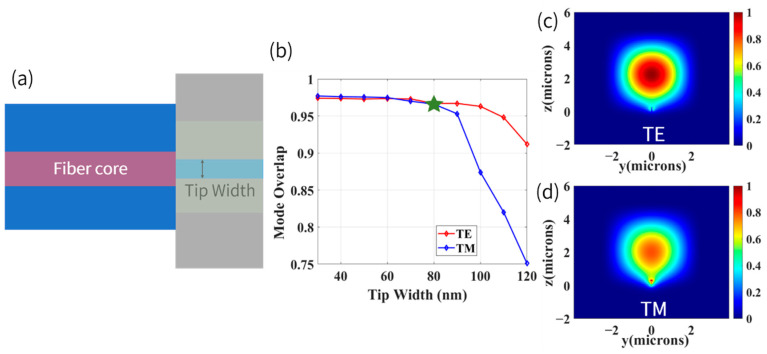
(**a**) Schematic of a setup for calculating mode overlap integral. (**b**) Mode overlap between tip and fiber with different tip widths (waveguide thickness = 260 nm). (**c**) Mode field distribution of TE mode at the tip end of inverse taper when the tip width was 80 nm. (**d**) TM mode.

The mode converter is capable of transforming fundamental modes of broad waveguide into fundamental modes of strip waveguide, and inverse taper is deemed as an excellent candidate to realize coupling between strip waveguide and lensed fiber [62]. The coupling efficiency of an inverse taper for edge coupler is primarily governed by two factors: inverse taper shape design and tip width selection. The shape design of inverse taper should be judiciously carried out to efficiently steer the light from a single-mode strip waveguide to tip waveguide at the end of inverse taper, while tip width should be selected appropriately to achieve superior mode overlap with fiber. A proper value for tip width is initially explored by calculating mode overlap integral between the chip facet near the tip of the inverse taper and fiber, as illustrated in Figure 7a. The mode overlap as a function of tip width is shown in Figure 7b. It is evident that when the tip width exceeds 80 nm, the mode overlap of TE and TM will decrease significantly, and the mode overlap of TM is more sensitive to the tip width. The difference between TE and TM mode is induced by the fact that polarization of the TE mode is parallel to the direction of taper width, which varies gradually to 120 nm, while polarization of TM mode is parallel to the direction of taper thickness, which is a constant value (260 nm). In this condition, when the tip width is increasing, the confinement degree of inverse taper for TM mode becomes large than TE mode, that is to say, the delocalization of TM mode becomes weaker than TE mode [63], which means TM mode has a smaller mode profile, as shown in Figure 7c,d. To achieve efficient coupling for both TE and TM modes while thinking over convenience of fabrication, the tip width was chosen to be 80 nm, which results in approximately 96.7% mode overlap integral for both TE and TM modes (marked with a green star in Figure 7b).

**Figure 8:**
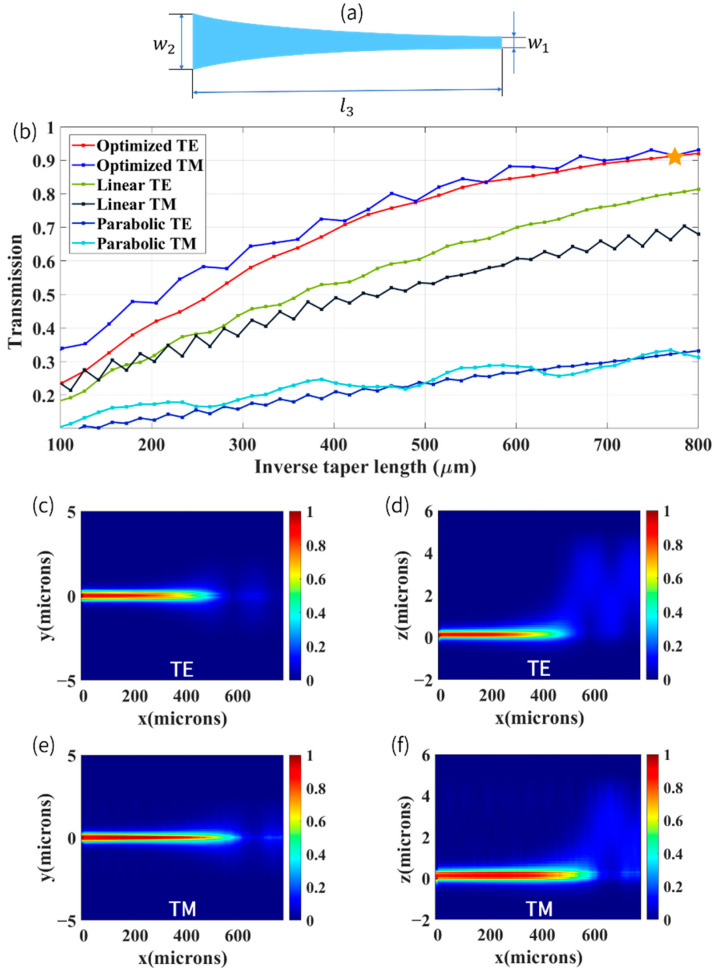
(**a**) Top view of optimized inverse taper. (**b**) Transmission as a function of inverse taper length for different shape design connecting an 80 nm width tip waveguide and 500 nm width strip waveguide. (**c**) Top view of simulated electric field evolution along the inverse taper, TE mode. (**d**) Side view of electric field, TE mode. (**e**) Top view, TM mode. (**f**) Side view, TM mode.

Figure 8a shows the schematic shape design of the proposed inverse taper, where width was tapered from 500 nm ($w_{2}$) down to a tip end 80 nm ($w_{1}$) to expand guided mode. From Figure 8b, we can see that with the increase in inverse taper length ($l_{3}$), the transmission (i.e., coupling efficiency) both for TE and TM modes ascended gradually for the cases of parabolic, linear, and optimized profile used in our work. It can be clearly seen that the transmission of the parabolic inverse taper was low. The fabrication of the linear inverse taper was straightforward and convenient, but its drawback was that the coupling efficiency of the TE and TM modes was quite different. Ren et al. drew conclusions that device size, fabrication intolerance, and misalignment tolerance of quadratic inverse taper were outstanding; more importantly, that the quadratic inverse taper had little difference in transmission for fundamental TE and TM modes [63]. Therefore, to achieve efficient transmission of TE and TM modes simultaneously, a nearly quadratic taper was designed, as described in Equation (13):

(13) $$W(l)=\rho {\left(l_{3}-l\right)}^{2.08}+w_{1},\rho =(w_{2}-w_{1})/l_{3}{}^{2.08}$$

where W is width of the optimized inverse taper, and l is length of optimized inverse taper from wide section. As can be seen from Figure 8b, compared with other two inverse tapers, this optimized inverse taper can improve the conversion efficiency both for TE and TM modes obviously and can achieve coupling efficiency of about 91.7% simultaneously when the length of the inverse taper was equal to 774.1 μm (marked with the orange star in the Figure 8b). For our optimized inverse taper, if the taper length was larger than 800 μm, the transmission almost became stable, which was because of the fact that the spot size evolution did not change greatly and in turn led to steady transmission. To evaluate and characterize the mode conversion performance of our optimized inverse taper, a side view and top view of the electric field propagation for TE and TM modes were carried out to observe the mode profile transition along the inverse taper, as shown in Figure 8c–f. It can be seen that as the width of the waveguide decreased, the expanded mode was re-confined in the polymer waveguide to achieve efficient fiber–chip coupling.

**Figure 9:**
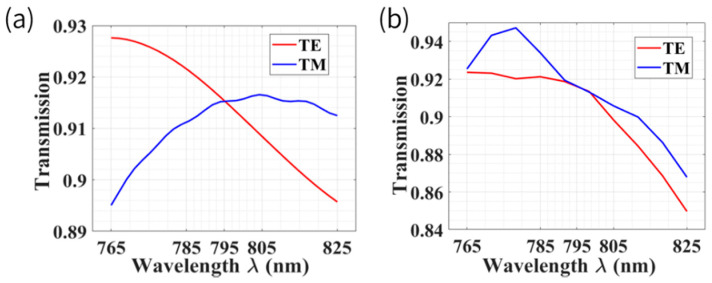
(**a**) Spectral response of the proposed mode converter with a length of 121.5 μm. (**b**) Spectral response of the optimized inverse taper with a length of 774.1 μm.

Figure 9 illustrates the operation bandwidth of the mode converter and optimized inverse taper in our study. From Figure 9a, we can see that for the mode converter, the coupling efficiency of TE and TM modes was still able to maintain more than 89% in the 60 nm (from 765 nm to 825 nm) range, and for the inverse taper, it was able to maintain more than 85% coupling efficiency, as shown in Figure 9b, which means that the fiber–chip spot size converter proposed in this paper is suitable for several chip-scale AMs (e.g., Rb, K). In addition, as for double beam Cs AMs [42,64,65,66], due to D1 and D2 lines (894 nm and 852 nm, respectively) of Cs being spectrally well separated, we are able to take advantage of the wavelength-dependent efficiency of this device. Specifically, such a grating-based polarimeter with a narrow bandwidth operating at D2 line of Cs could be designed judiciously, and coupling efficiency is almost zero at D1 line of Cs. This is a distinct advantage of such an integrated device compared with traditional and bulk PBS, because when dual co-propagating beams are utilized to pump and probe, it enables both filtering and differential optical rotation angle measurement simultaneously on chip.

**Figure 10:**
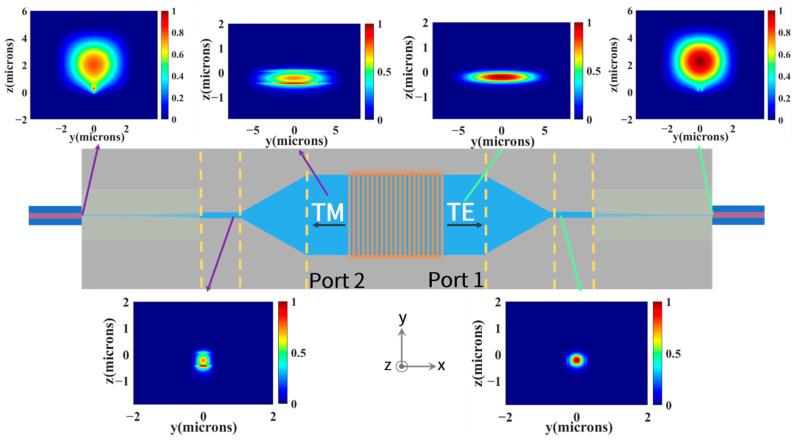
Mode field distribution at different positions of the polarization-splitting grating coupler with horizontal integration. The planar waveguide modes on Port 1 and Port 2 were located and converted to single-mode strip waveguide, and then efficiently coupled into lensed fibers through the optimized inverse taper.

Mode evolution of the proposed polarization-splitting grating coupler with horizontal integration is shown in Figure 10. When the y-polarized incident light propagates to the polarization-splitting grating coupler, it will be coupled to the TE mode of planar waveguide on Port 1, as mentioned earlier; then converts to fundamental TE mode of strip waveguide by mode converter; and finally achieves efficient fiber–chip coupling with the help of the optimized inverse taper. When the incident light is x-polarized, it will be coupled to the TM mode on Port 2 and eventually be coupled into lensed fiber similarly. In summary, when the linearly polarized probe light that experienced optical rotation due to magnetically induced circular birefringence of Rb illuminates the structure, orthogonal linear-polarized components (x-polarized and y-polarized) can be split and coupled into the corresponding two lensed fibers with a coupling efficiency of 26.9% simultaneously. The optical rotation angle of linearly polarized probe light can be accurately measured by means of the differential detection method, and the unknown magnetic field can be subsequently obtained.

### 3.3. Application on Chip-Scale Atomic Magnetometer

**Figure 11:**
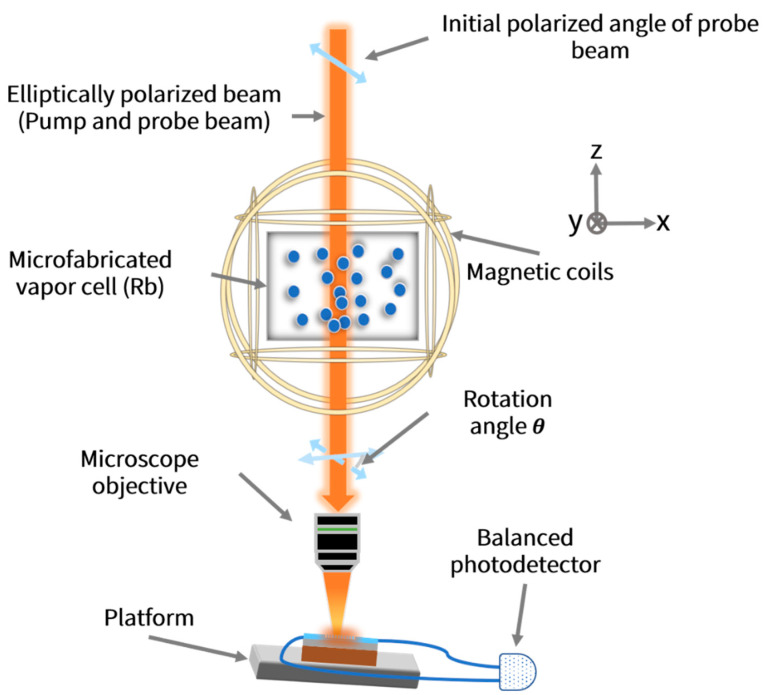
Schematic sketch of chip-scale AM demonstrated in this study according to the polarization splitting grating coupler with horizontal integration.

A schematic sketch of a chip-scale AM based on the polarization-splitting grating coupler with horizontal integration is demonstrated in Figure 11. A single elliptically polarized beam that is nearly resonant with transition center (detuned by 50 GHz from ^87^Rb D1 line) was employed. The circularly polarized component was utilized to achieve optical pumping, while the linearly polarized component was utilized to realize optical rotation detection [48]. The procedure of magnetic field measurement can be briefly summarized as follows: firstly, the circularly polarized component of incident light polarizes Rb by optical pumping, which can substantially increase the optical rotation angle [67]. Secondly, the custom Helmholtz magnetic coils are utilized to minimize ambient magnetic field, and then a small static magnetic field $B_{x}$ and an oscillating modulation field $B_{mod}$ are generated. Thirdly, the transmitted light with optical rotation is focused on the coupler using microscope objective with large magnification to make spot size match. Eventually, the light coupled to Port 1 and Port 2 is collected to the balanced photodetector making use of a pair of lensed fibers.

In this paper, single-mode input of the polarization-splitting grating coupler was reasonably assumed (see Supplementary Materials and Figure S1 for details). However, it is possible that there is higher-order mode excitation in the grating region due to manufacturing and packaging errors. Multimodality of input mode is a non-negligible interference to experimental work (see Supplementary Materials for details), which may result in imbalance in polarization measurements of the transmitted probe beam. To overcome such an obstacle, the output lensed fibers fixed to Port 1 and Port 2 should be combined with variable attenuator fibers prior to connecting to the balanced photodetector during experiment tests. By adjusting the attenuator fibers judiciously, a difference in coupling efficiency between Port 1 and Port 2 due to multimodality of input could be appropriately compensated. In absence of external magnetic field input, the output voltage of the balanced photodetector is zero. The normalized differential signal is shown as follows:

(14) $$S_{diff}=S_{Port1}-S_{Port2}\propto {\left(E_{y}^{C}\right)}^{2}+{\left(E_{y}^{L}\right)}^{2}-{\left(E_{x}^{C}\right)}^{2}-{\left(E_{x}^{L}\right)}^{2}={\left(E_{y}^{L}\right)}^{2}-{\left(E_{x}^{L}\right)}^{2}$$

where ${\left(E_{x}^{C}\right)}^{2}$ and ${\left(E_{y}^{C}\right)}^{2}$ are orthogonal components of the circularly polarized component of incident light, and ${\left(E_{x}^{L}\right)}^{2}$ and ${\left(E_{y}^{L}\right)}^{2}$ are of the linearly polarized component. The two components ${\left(E_{x}^{C}\right)}^{2}$ and ${\left(E_{y}^{C}\right)}^{2}$ are equal since the light for optical pumping is circularly polarized. The relationship between the normalized differential signal $S_{diff}$ and external magnetic field $B_{x}$ is expressed by Equation (15), where external magnetic field $B_{x}$ is in an approximate linear range of the Lorentzian profile.

(15) $$S_{diff}(B_{x})\propto \sin\theta =\sin[\kappa \frac{\gamma B_{x}}{{\left(\gamma B_{x}\right)}^{2}+R^{2}}]$$

As a result, compared to AMs that utilize traditional polarization beam splitter to measure optical rotation angle [47,48], chip-scale AM (single elliptically polarized beam, SERF regime, ^87^Rb, modulated field), which harnesses the planar integrated photonics device demonstrated in this paper, has the potential to achieve differential measurement of the external magnetic field within the small near-zero-field range accurately on a chip while dramatically reducing the volume of the current miniaturized AM, which facilitates the development of high spatial resolution MEG and MCG. More importantly, this integrated balanced-polarmetric device overcome the drawbacks that most current miniaturized AMs are not capable of achieving in terms of optical rotation angle differential detection. Indeed, this integrated device could find application in different kinds of AMs such as perpendicular double-beam AMs.

## 4. Conclusions

In summary, a polarization-splitting grating coupler with horizontal integration based on chip-scale atomic magnetometer (AM) application is proposed. This device enables efficient coupling and polarization-splitting of two orthogonal linear-polarized lights simultaneously in the D1 transition wavelength of Rb. In other words, high-efficiency differential measurement of magnetic field was achieved on chip in this paper, overcoming the drawback of the fact that current miniaturized AMs are not capable of realizing optical rotation detection. Compared with conventional bulk polarization beam splitter (PBS), such an integrated device provides two revolutionary advantages. Firstly, bulk PBS occupies a relatively large volume, hindering further miniaturization and integration of AM. The integrated device replaces tradition PBS through a simple, more easily integrated grating structure whilst dramatically reducing the volume of AM. Secondly, conventional components require precise machining and complex alignment, which hampers us from obtaining inexpensive and robust AM. In virtue of current CMOS-compatible micro/nano-fabrication technology, the integrated device could be mass-produced at a low cost. Moreover, with the help of advanced photonics packaging technology, complex alignment of discrete bulk optical components could be avoided, and the robustness of miniaturized AM could be enhanced dramatically. In addition, from the perspective of integrated devices, compared with the PSS method declared recently [49], our structure has a smaller spot size mismatch so that higher coupling efficiency can be acquired. In addition, materials applied and the fabrication process of the grating coupler is compatible with current CMOS-compatible microfabrication technology, and therefore compared with metasurface-based nanophotonics components [50] that apply ultra-small features and non-transparent materials, our device is more favorable for massive production. Incident light of chip-scale AM demonstrated in this paper was tilted with a small angle (4°), which is a common practice that can be realized by bonding with optical phase arrays (OPAs) [68] on the bottom of microfabricated vapor cell in future.

Nowadays, chip integration has become a major demand for atomic magnetometers, being challenging to achieve because of traditional and bulky optical components. Therefore, the combination of atomic devices and integrated photonics technologies has recently garnered increasing attention. In addition to the polarization-splitting grating coupler with horizontal integration demonstrated in this study, metalens [69] is promising in replacing the traditional microscope objective and is compatible with planar integration. However, it should be emphasized that improving coupling efficiency of such an integrated device could lead to an enhancement of signal-to-noise ratio and promote sensitivity of chip-scale AM. Further improvement on coupling efficiency of grating coupler is realizable with the help of bottom metal mirror [70], apodized grating [71], and inverse design methods such as intelligent algorithms [72] and deep neural networks (DNNs) [73]. As for mode converter, multi-stage [74] or adiabatic taper [75,76] are beneficial to improvement of conversion efficiency. Multi-tip [77,78] or multi-layer [79] structures are capable of enhancing coupling efficiency from photonic chip to lensed fibers whilst making the device ultra-compact. We will implement on-chip integration of traditional optical components widely utilized in conventional miniaturized AM while achieving comparable performance in future work. Furthermore, integrated silicon-compatible photodetectors will be designed and combined with such a polarization-splitting grating coupler on chip to take a significant step towards chip-scale AMs. This paper provides a new method that is deemed to be a promising solution for realizing chip-integrated AMs, undoubtedly breaking through the limitations of configurations of traditional AMs and facilitating the developments of multi-channel bio-magnetic imaging.

## Data Availability

The data presented in this study are available on request from the corresponding author.

## Supplementary Materials

The following supporting information can be downloaded at: https://www.mdpi.com/article/10.3390/bios12070529/s1, Figure S1: Normalized mode proportion of TE modes in the polarization-splitting grating coupler at Port 1. Figure S2: Transmission of the high-order modes (TE1 and TE2) of high-aspect ratio waveguide to fundamental mode (TE0) of single mode strip waveguide as a function of length of the mode converter (wavelength equals to 795 nm).
