# Supplementary material for: Integrated Polarization-Splitting Grating Coupler for Chip-Scale Atomic Magnetometer
Source: Biosensors (Basel). 2022 Jul 15;12(7):529. doi: 10.3390/bios12070529 (PMC9313279; doi:10.3390/bios12070529)
Supplement: Supplementary file 1 [file biosensors-12-00529-s001.zip › biosensors-1789994-supplementary.pdf]

Supplementary material

Integrated polarization-splitting grating coupler for chip-scale atomic magnetometer

Jinsheng Hu [1,2], Jixi Lu [2,3,*], Zihua Liang [1,2], Lu Liu [2,3], Weiyi Wang [2,3], Peng Zhou [1,2], Mao Ye [2,3]

1 School of Instrument and Optoelectronic Engineering, Beihang University, Beijing 100191, China;
2 Beihang Hangzhou Innovation Institute Yuhang, Xixi Octagon City, Yuhang District, Hangzhou 310023, China;
3 Research Institute for Frontier Science, Beihang University, Beijing 100191, China
* Correspondence: lujixi@buaa.edu.cn (J.L.)

1. Multimodality analysis of input mode

1.1. Evaluation of multimodality of input mode

Three-dimensional finite-difference-time-domain (3D-FDTD) simulation of the polarization-splitting grating coupler has been performed, assuming that the incident light is y-polarized and appropriate tilted angle and position are chosen as described in main text. In this condition, TE modes of high-aspect ratio waveguide are excited at Port 1. To evaluate multimodality of input mode, we added a Mode Expansion Monitor (Lumerical Mode Solutions, Inc.) at Port 1 and monitored TE0 (fundamental TE mode), TE1, TE2, TE3, TE4 (both of them are higher-order modes) excitation proportion as shown in Figure S1. Obviously, the modes input to the grating PBS are almost all fundamental mode (proportion is about 99%), the remaining proportion of higher-order modes (TE1, TE2, TE3, TE4) is about 1%. If the incident light is x-polarized, the excitation of TM mode is similar at Port 2. Therefore, single-mode input can be assumed reasonably in this paper.

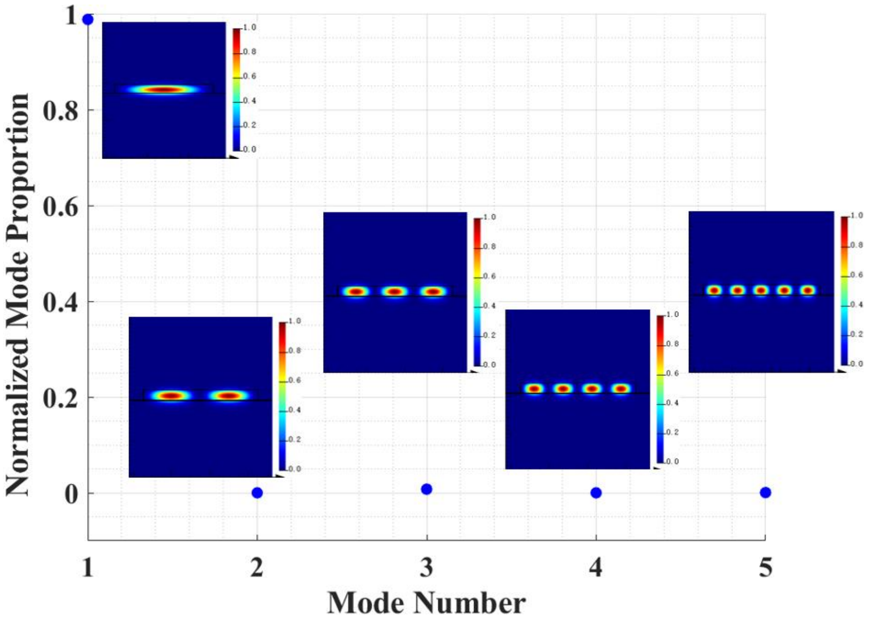

Figure S1. Normalized mode proportion of TE modes in the polarization-splitting grating coupler at Port 1.

1.2. Interference of experimental work due to multimodality of input mode

As a matter of fact, the effective refractive index (in other words, propagation wave vector) of fundamental mode (such as TE0 mode) is very similar with higher-order mode (such as TE1 or TE2 modes) in the wide waveguide grating coupling region (its width is 12 μm in our paper). Consequently, even if we design the polarization-splitting grating

coupler judiciously according to the effective refractive index of the fundamental mode, it is possible that there are higher-order modes excitation in the grating region due to manufacturing (such as deposition, lithography or etching) and packaging errors. In order to quantify the impact of multimodality input mode on results. Simulation of mode converter has been performed under the condition of multi-mode input. Figure S2 depicts the transmission of mode converter from higher-order modes conversion (such as TE1 and TE2) of high-aspect ratio waveguide to fundamental mode (TE0) of single mode strip waveguide with wavelength equals to 795 nm. For TE1 to TE0, it is obviously shown that with the increase of converter length, the transmission decreases drastically, the transmission drops to 7.8% when length of converter equals to 121.5 μm (chosen in this paper, 91.5% transmission for fundamental TE and TM mode simultaneously). For TE2 to TE0, there is hardly any mode conversion process. When the input modes are higher-order TM modes, the conversion results are similar.

In this paper, the ultimate aim is generation of a balanced-polarimetric signal, which is the foundation of optical rotation angle differential detection (i.e., magnetic field differential detection). From the simulation result mentioned above, we are able to draw the conclusion that the multimodality of input mode is a non-negligible interference to experimental work, which may lead to imbalance in polarization measurements. To overcome such an obstacle, the output lensed fibers fixed to Port 1 and Port 2 should joint variable attenuator fibers before connecting to commercial balanced photodetector. By adjusting the attenuator cautiously, difference in coupling efficiency between Port 1 and Port 2 due to multimodality input mode could be compensated properly.

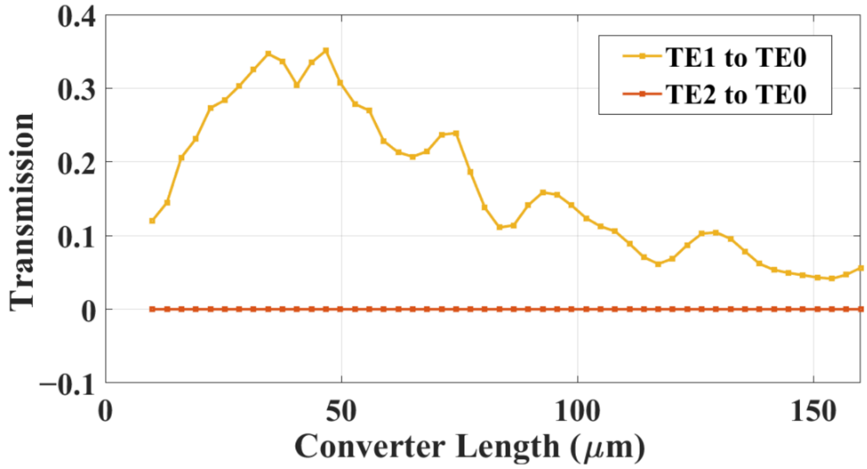

Figure S2. Transmission of the high-order modes (TE1 and TE2) of high-aspect ratio waveguide to fundamental mode (TE0) of single mode strip waveguide as a function of length of the mode converter (wavelength equals to 795 nm).
